# CRISPR: a journey of gene-editing based medicine

**DOI:** 10.1007/s13258-020-01002-x

**Published:** 2020-10-22

**Authors:** Zhabiz Golkar

**Affiliations:** Division of Academic Affairs, Voorhees College, Denmark, SC USA

**Keywords:** CRISPR, Gene-editing, *Cas*, Bacteria, Adaptive immunity, Medicine, DNA, Ethic

## Abstract

CRISPR (Clustered Regularly Interspaced Short Palindromic Repeat) is one of the hallmark of biological tools, contemplated as a valid and hopeful alternatives to genome editing. Advancements in CRISPR-based technologies have empowered scientists with an editing kit that allows them to employ their knowledge for deleting, replacing and lately “Gene Surgery”, and provides unique control over genes in broad range of species, and presumably in humans. These fast-growing technologies have high strength and flexibility and are becoming an adaptable tool with implementations that are altering organism’s genome and easily used for chromatin manipulation. In addition to the popularity of CRISPR in genome engineering and modern biology, this major tool authorizes breakthrough discoveries and methodological advancements in science. As scientists are developing new types of experiments, some of the applications are raising questions about what CRISPR can enable. The results of evidence-based research strongly suggest that CRISPR is becoming a practical tool for genome-engineering and to create genetically modified eukaryotes, which is needed to establish guidelines on new regulatory concerns for scientific communities.

## Introduction

**Fig. 1:**
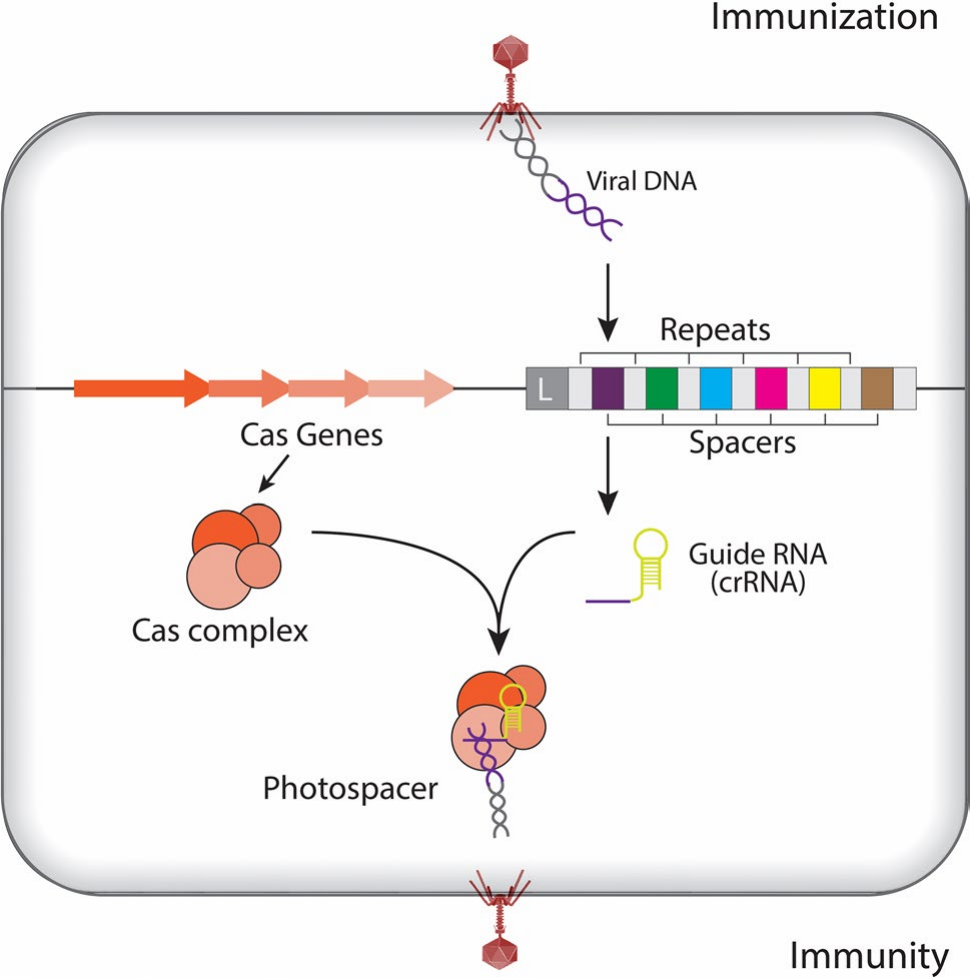
Overview of the CRISPR-Cas9 system. CRISPR loci are found in roughly 40% of bacterial genomes and can be transmitted both horizontally and vertically

It has been well documented that phages overcome bacterial resistance by reverting their host genomes. Therefore, a large segments of the bacterial DNA is occupied by the transplanted encoding genes from different antiviral defense systems (Brouns et al. 2008; Lintner et al. 2011; Weekes and Yuksel 2004; Wiedenheft and Van-Duijn 2011). Upon infection and completion of phages replication, prophages destroy the host cell and to avoid this lethal threat, bacteria evolve various phage-resistance defense system that obstruct almost each phase of phage life cycles. Most bacteria, alter their existing receptors on cell membrane to restrict natural viral attachment using restriction enzymes to destroy invaded viral DNA once infects the cell. Bacteria have adapted an altruistic suicide strategy to inhibit propagation of viral DNA, within their population. Overall, such anti-viral mechanisms frequently provide enhanced protection from the interference of analogous genetic assaults, DNA molecules, plasmids, and other conjugative and integrative components (or elements) (Cong et al. 2013; Almendros et al. 2014; Horvath and Barrangou 2010). A classical model of this coevolution is the *E. coli* restriction-modification system as it relates to the counter-attack interactions of the bacterial host against T4 bacteriophages (Zhu and Ye 2015; Chylinski et al. 2014; Makarova et al. 2011; Deltcheva et al. 2011; Sapranauskas et al. 2011). This Darwinian interchange between majority of prokaryotes in focus on bacteria and viruses stimulates an inevitable evolution in expression of bacterial phage-resistance system (Hale et al. 2010). These unique restriction-modification systems in bacteria are not only able to discriminate “self” vs. “non-self” DNA, but also act as a primitive innate immune system that confers resistance against invasive DNA. This adaptive microbial immune system has a genomic etiology, which has been coined as CRISPR, and it delivers acquired immunity against invading viruses and plasmids in host cells. CRISPR consists of between 20 and 49 base pair long and highly conserved short DNA repeat sequences which are at least partially palindromic (Rath et al. 2015). These repeats are interspaced by stretches of variable sequences of between 24 and 75 base pair spacers (Raz and Tannenbaum 2010; Marraffini and Sontheimer 2008). The spacer sequences usually originate from (1) fragments of captured foreign DNA; (2) coding or non-coding DNA; or (3) DNA derivative of RNA from various viruses, phages, transposons or plasmids (Ferguson et al. 2011; Golkar 2016) (Fig. 1).

While, CRISPR technology and its revolution seem to occur at a truly astonishing pace, this prokaryotic immune system is especially flexible for genome engineering, thus offering more precise on-target and decreasing off-target editing rates. Prokaryotes have utilized CRISPR as an adaptive immune system against viral attacks and have long like a winning strategy to ensure prokaryotic survival. This system is proving to be just as powerful for research studies, outshining existing genome editing research applications such as ZFNs and TALENs (Bondy-Denomy et al. 2013; Bondy-Denomy 2018). The development and understanding of the CRISPR/Cas and bacterial immune system and its application into a gene-editing tool are the efforts of several researchers over long period of time. This review offers a brief history of each significant contribution of the researchers who were involved in this discovery and shaped the CRISPR gene editing technology to its clinical application thus crossing boundaries by gene surgery in human embryo (Broad Institute 2018).

## Discovery of CRISPR and its function

In 1987, Yoshizumi Ishino discovered the CRISPR sequences in *E. coli* genome (Ishino et al. 1987) and extensive analysis of gene coding for isozyme conversion of alkaline phosphatase was reported by him later on in 2018 (Ishino et al. 2018). However, their function as a defense mechanism against phages was not discovered until 2007. In 1993, Francisco Mojica from the University of Alicante, Spain was the first scientist to elucidate CRISPR locus, and he reported the structures of the currently known CRISPR sequences in 2000 (Mojica et al. 2000) He further characterized some of CRISPR sequences that have homology with genome of bacteriophages, and he reported that CRISPR is a unique form of bacterial adaptive immune system in 2005 (Mojica et al. 2005). In 2005 Alexander Bolotin discovered set of *Cas* genes, that coding for a large functioning protein that projected to have nucleus activity, later named as *Cas9* in CRISPR locus of *S. thermophilus*. His team noticed that all spacers with conserved similarity to bacteriophage genes share a unique sequence at one end, now known as Protospacer Adjacent Motif (PAM) which is necessary for target recognition (Bolotin et al. 2005; Jansen et al. 2002). In 2006, Eugene Koonin demonstrated a fundamental structure for CRISPR cascades based on insertions of homologous sequences to bacteriophage genome in the spacer array (Koonin and Makarova 2019; Makarova et al. 2006). In 2007, Barrangou et al. (2007) demonstrated that consistent exposure of targeted bacteria to bacteriophage attack may led to resistance; that viral DNA will be inserted into bacterial CRISPR interspacing regions; and that if these viral DNA are removed from the spacers, targeted bacterial will be sensitive to viral attack and lose resistance. Furthermore, Philippe Horvath performed the first experiment with CRISPR systems in *S. thermophilus* by insertion of new sequence of phage DNA into the CRISPR array, and he studied the immunity of bacteria against the next round of phage attack. He demonstrated that *Cas9* protein is only required for interference, while CRISPR cascade inactivates phage attacks (Horvath and Barrangou 2010). In 2008, John van der Oost and his colleagues from the University of Wageningen, Netherlands confirmed the presence of CRISPR RNAs (crRNAs) spacer sequences in *E. coli* genome. These spacers are originated from viral DNA and are transcribed into crRNAs, which directly target the DNA after activation of *Cas* protein (Lander 2016). Marraffini and Sontheimer (2008) further confirmed that the concept of target molecule is always DNA not RNA, and that the CRISPR system is a unique tool that could be applicable to the eukaryotic system. Later, in 2009, Hale and colleagues discovered different types of CRISPR that could exclusively target RNA (Hale et al. 2009; Garneau et al. 2010). In 2010, S. Moineau introduced Type II of CRISPR systems, which demonstrated that in CRISPR-Cas9’ system, the *Cas9* protein, in conjunction with crRNAs, is required to cleavage the target DNA in 3 nucleotides upstream of the PAM and in very specific positions (Deveau et al. 2010; Jinek et al. 2012). In 2011, E. Charpentier and team (Deltcheva et al. 2011) discovered CRISPR RNA (tracrRNA) while performing RNA sequencing on *S. pyogenes,* which has a *Cas9* protein. She presented that tracrRNA creates a duplex with CRISPR RNA (crRNA), which guides *Cas9* to its targets (Abbott 2016). Meanwhile, Siksnys and colleagues cloned Type II system of CRISPR-*Cas* loci of *S. thermophilus* and expressed that in *E. coli* the result was the ability of this strain to provide plasmid resistance. This experiments proved that CRISPR systems are independent functioning sequences (Sapranauskas et al. 2011). In 2012, Jinek et al. (2012) first in vitro experiment showed, CRISPR could be designed for targeted DNA cleavage. In 2013, first application of CRISPR based editing in human and mouse cell lines was reported by Cong et al. (2013) and Mali et al. (2013). Finally, one of the pioneers of TALENs, Zhang, could modified CRISPR-*Cas9* for genome editing in human and mouse cell lines. They used two different *Cas9* proteins (*S. thermophilus* and *S. pyogenes*) and performed cleavage in targeted DNA of these eukaryotic cells. Their experiments show it is possible to edit CRISPR-Cas system and use it for different targets of the same genome (Cong et al. 2013). Before 2018, PubMed had published over 5000 CRISPR-*Cas* articles, several of publications focused on improvement of tool’s specificity, orthogonality, accuracy, applications and multi-plexibility in diverse organisms.

The results of the studies elucidated that extreme diversity in CRISPR mechanism, found in 95% of archaeal and 48% of bacterial genomes with respect to unique PAM sequences and types/subtypes of *Cas* proteins.

**Fig. 2:**
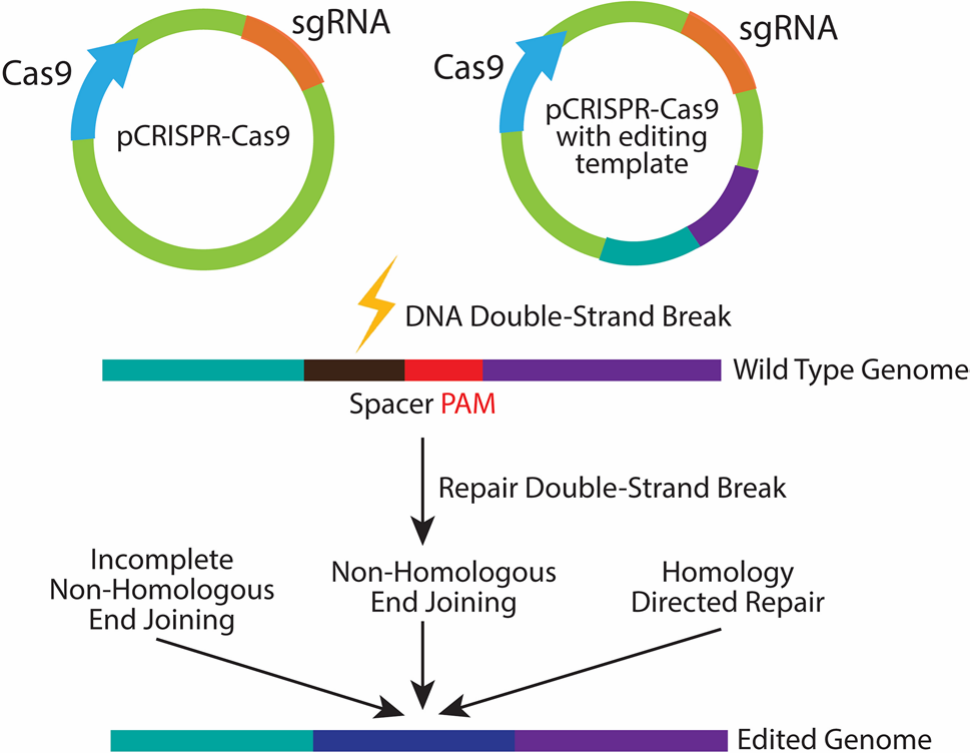
CRISPR-Cas9 as a precise genome editing tool

In 2011, Makarova et al. (2011) identified 5 major types and 16 subtypes based on shared characteristics, evolutionary and sequence homology. He further classified them into two classes based on the structure of the effector complex that cleaves the genomic DNA (Makarova et al. 2011). The CRISPR might be a powerful defense system to ensure prokaryotic survival, but it is not invincible. In 2012 scientists (Cady et al. 2012) reported the first set of anti-CRISPR genes in phages attacking *Ps. aeruginosa* that can block CRISPR function, inhibit the activation of *Cas* proteins or prevent the CRISPR-Cas system from binding to target DNA (Borges et al. 2017). Currently, CRISPR tool advancement has arisen at a surprising pace, with research focused towards enhancing on-target and reducing off-target editing rates. Biotech companies have empowered researchers to improve further the CRISPR technique by providing constructed plasmids, and have shared the experiences of other scientists, and have created a tremendous access to numerous plasmids applicable in many platform applications and in a variety of models e.g.; eukaryotes (Human, mouse, and rat); Prokaryotes (*E. coli, Streptococcus, Streptomyces, and others*), Drosophila; Plants (monocots and dicots); *C. elegans*; Yeast (*S. cerevisiae and S. pombe*); Zebrafish, and Xenopus (Adli 2018; Wang et al. 2014a, b; Koike-Yusa et al. 2014; Pourcel et al. 2005). More than a decade passed before scientists discovered the landscape of these spacer sequences. Computational analysis of genomic sequences of several organisms, including bacteriophages, led researchers to notice the importance of CRISPR repeat and spacer sequences. Conspicuously, CRISPR had been identified as an atypical bacterial DNA repeat element for many years before it was reported as the bacterial immune system and subsequently introduced as a potent re-programmable gene-targeting tool kit (Adli 2018; Wang et al. 2014a, b; Koike-Yusa et al. 2014; Pourcel et al. 2005) (Fig. 2).

## The rise of CRISPR as the genome engineering application

While the breakthrough in synthetically designed, mega nucleases enzymes using ZFNs and TALENs sequentially enhanced the gene-editing efficiency, targeting various recognition sites in the genomic DNA requires more new proteins to be re-designed. The major challenge in further engineering new proteins in ZFNs or TALENs is who are not applying these techniques as frequently as CRISPR has revolutionized the field, and provides a simpler and more fixable kit for gene- editing efficiency. However, use of any of gene-editing tool depends on the application, type of cell lines, and research goal. This discovery, in addition to the next generation of sequencing, provides a powerful tool kit for scientists to target multiple locus on DNA of the same eukaryotes or prokaryotes and genome-wide screening. The combination of gRNA targeting can be applied to identify important genes which code for a certain phenotype. A substantial pooled of information is present for gene knockout, transcriptional activation or repression in cells (Brophy and Voigt 2014; Jusiak et al. 2016). As CRISPR technology is developing, many researchers are interested in exploring further the implication and functioning of these CRISPR sequences. Findings are notably followed by groundbreaking literatures published worldwide demonstrating that CRISPR could be tailored for various gene alteration in vivo in both prokaryotes and mammalians (Brophy and Voigt 2014; Rothschild et al. 2015). CRISPR has provided an especially practical and accessible tool that could be simply pointed to a selected location in the genome by designing a short sgRNA sequence. In past few years, utilizing the CRISPR/Cas9 tool has exceed the basic genome editing and targeting DNA. In some of the current approaches CRISPR applications have been used in chromatin (Adli 2018), and targeted epigenetic regulation, precision of CRISPR to achieve large-scale functional screenings by using guided proteins with thousands of copies of sgRNA in each targeted cell to recognize genes that influence an explicit phenotype in an individual (Lino et al. 2018). These approaches need several technical and analytical improvements, (Dar et al. 2018) but once recognized as standard approaches, they could be powerful tools to monitor the functionality of large numbers of genes simultaneously. The specificity and in-depth details of these assays are not in scope of this article however in 2018, Dar et al. (2018) and Adli (2018) have published their excellent analysis in wide range of CRISPR screening and well documented in their articles.

## Examples of the use of the CRISPR-Cas in modern medicine

Regulation of cell behavior is one of the major goals of synthetic biology, and to achieve this goal requires efficient and precise artificial transcription factors that could target the specific sequences. CRISPR system has been used to adjust processes in a different prokaryote and eukaryotic cells. These regulatory pathways have been established using transcriptional regulatory systems based on CRISPR-*Cas9*. Considering CRISPR has made the great progress in transcriptional regulation, this allows several genes to be set simultaneously (Hasty and Montagna 2014; Gerlinger et al. 2014). Recently, the development of nuclease-dead *Cas* molecules (dCas9 and dCas12a) as a new comprehensive toolbox has offered an additional platform for scientists to regulate the genome function and to control cellular behaviors without using CRISPR for genome editing (Xu and Qi 2019). Chronic granulomatous disease is caused by a single intronic mutation in the CYBB gene. Flynn et al. (2015), have used CRISPR/Cas9D10A, nickase and donor-mediated HR in iPS of skin fibroblast for In vitro differentiated macrophage function. Barth syndrome is a result of 1 bp deletion on chromosome. Wang et al. (2014a, b) have used iPS of skin fibroblast and applied CRISPR/CAS9, PiggyBac and donor-mediated HR for In vitro differentiated cardiomyocyte and muscle contraction. Similarly, β-Thalassemia is A/G and TCTT deletion or C > T mutation in HBB gene, and studies have shown that modification of CRISPR/CAS9, PiggyBac, and donor-mediated HR using iPS fibroblast could be used for In vitro hematopoietic differentiation and Gene expression (Xu et al. 2015; Xie et al. 2014). In another study on Hemophilia A conducted by Park et al., researchers used iPS; urine cells of Hemophilia A patients and Cas9 protein, gRNA and DNA plasmid were transferred using a microporator system. The results were demonstrated in vivo differentiated endothelial cells and transplanted into a hemophilia mouse for correction of gene inversion 140 kb and 160 kb from intron 1–22 (Park et al. 2015). Cystic fibrosis is another mutation-based condition on CFTR F508 deletion, CRISPR/CAS9 and donor-mediated HR, using no iPS cells. Only 3D intestinal organ culture has shown interesting results for In vitro differentiated intestinal organoids (Schwank et al. 2013; Hastings et al. 2009). In 2019, report has shown promise in a clinical trial application in patients with Sickle cell anemia (Humbert et al. 2019), hematopoietic stem cells of patient was targeted for editing. Researchers used CRISPR-based technique to edit the antigens of CD90 and boost the regenerating this cells with normal function in blood.

## Application of CRISPR as a tool to treat Duchenne Muscular Dystrophy in mice

In 2017 Bengtsson and colleagues used CRISPR genome-editing tools to treat Duchenne Muscular Dystrophy (DMD) in mice (Bengtsson et al. 2017). In Duchenne Muscular Dystrophy, defective DMD gene is unable to code for the protein dystrophin, and there is no known cure for DMD. Bengtsson et al., used CRISPR to remove the defective DMD gene in mice, which has allowed the mice to produce major dystrophin proteins in the muscles cells. The results of the study were promising and considered as the first application of CRISPR in the treatment of genetic disease in mice with DMD. CRISPR can delete the incorrect exon, therefore the duplicator system can generate a smaller amount of dystrophin protein as effective as its natural form and sgRNA. Modified *Cas9* were delivered by using unique viral career; adenovirus into mouse muscle cells; and CRISPR system was applied to delete the incorrect exon (Long et al. 2014; Nelson et al. 2016; Ousterout et al. 2015). In another study, the Li et al. (2015) group used iPS fibroblast and CRISPR/Cas9 for 75484 bp deletion, including exon 44 of Dystrophin gene, the results of which were used for in vitro differentiated skeletal muscle cells and evaluating the gene expression.

## CRISPR system and gene-editing in cancer

The CRISPR system has inclusive potential for a variety of applications in epigenetic cancer therapy, in gene regulation of oncogenes in cells, multiscale cancer modeling, proteomics methods for drug discovery and protein–protein interactions (PPI) as therapeutic targets in cancer focused studies (Dar et al. 2018; Sachdeva et al. 2015; Martínez et al. 2012), or chromosomal rearrangements in cancers that involve a single balanced fusion, or in combination with one or more fusions that disrupt this balance (Maresch et al. 2016). It has been studied in details cancer cells accumulate multiple mutations in genes that, can cause development of cancer cells, progression and distant metastasis (Shi et al. 2015; Weber et al. 2015; Sánchez-Rivera and Jacks 2015; Khan et al. 2016). The CRISPR system could possibly cure the disorders caused by these generating mutations by switching off the respective oncogenes or by switching on the tumor suppressor genes in activation or suppression of telomeres as another potential application in cancer therapy (Harley 2008; Annunziato et al. 2016; Torres-Ruiz and Rodriguez-Perales 2015; Yoo and Jones 2006; Chiba et al. 2015; Wang et al. 2017). In a study by oncologist Lu You, the T-cells were obtained from the patient’s blood of Non-small cell lung cancer and gene *PD* as a part of immune checkpoint were disabled by use of CRISPR. The *PD*-*1* gene naturally serves as an “off switch” to prevent T-cells from damaging normal cells, however several types of cancer cells seize the pathway to escape detection by the immune system and grow abandoned. In this prospective trial, the edited *PD*-*1* knockout T-cells were transferred back into the patient and were able to recognize and evade the Non-small cell lung cancer cell (You et al. 2019). The CRISPR/Cas9 advancement is also playing an important role in revolutionizing the current he diagnostic methods and available treatment for breast cancer (Yang et al. 2018). An invasive lobular breast carcinoma is taken as an example for this diagnosis and CRISPR/Cas9 mediated tool is used for modification of somatic cell in putative cancer driver genes. For therapeutic approaches, the inhibition of breast cancer cell proliferation is accomplished by adapting a dominant negative mutation generated by the CRISPR/Cas9-targetting of *HER 2* (Annunziato et al. 2016). Another promising application, T-cells of patient is extracting and after gene modification using CRIPSR, re-programmed cells infusing back into the patient to fight the Leukemia and Lymphoma cancer cells (Graham et al. 2018). Multiscale of cancer modelling is another application of the CRISPR system, via the stimulation of tumor suppressor genes and the suppression of oncogenes. Traditionally, in murinae cancer modelling, genetic modification of transgenes or homologous recombination in embryonic stem cells were required (Yao et al. 2015). These models could predict one or two mutations in the model and were often very expensive methods; (Jin and Li 2016) however, using the CRISPR system could generate the same information in less than a few weeks and was cost-effective (Nishimasu et al. 2014; Driehuis and Clevers 2017). Another application of the CRISPR system the use of epigenetic factors of some cancers.

Generally, methylation is a major epigenetic factor for gene expression and regulation in eukaryotic cells (Driehuis and Clevers 2017). CpG islands are typically located in the promoter region and during epigenetics events, methylation process will lead the repression of gene expression. DNA methylation in cells is extremely well regulated by a family of enzymes called DNMTs: DNMT1, DNMT2 DNMT3a and DNMT3b. If DNMTs are dysfunctional in cancer cells, they deactivate the cancer suppressor genes or activate oncogenes (Moore et al. 2013). The CRISPR system could target the desired genes and engineer their functions to generate mutations in enzymes associated with epigenetic regulation (Adli 2018).

Similarly, the CRISPR system has the potential to change the genome of somatic cells. In 2015, Singh et al., induced several organized mutations in a mice by using the CRISPR system concomitantly. Numerous parameters involved in the diverse types of cancers were predicted (Singh et al. 2015). Bak et al., used two lentiviruses associated with each other to express all the of CRISPR relevant factors in hematopoietic stem and progenitor cells (HSPC). The absence of functional mutations produced in these cells may led to acute myeloid leukaemia (AML) in mice (Heckl et al. 2014; Tothova et al. 2017). However, well-designed experiments in mouse of human genetic variants, e.g.; SNPs involved by genome-wide association (GWA) findings, have been thought-provoking. Currently, however, practically any change mirroring human coding variants could be initiated in the mice genome. The CRISPR system provided a platform of opportunities for specific genome engineering in humanized mice (Birling et al. 2017; Fujihara and Ikawa 2014). The CRISPR system equips mouse geneticists with a powerful, quicker, improved, and cheaper genetic toolbox, exclusively capable of testing human disease-related issues in mouse research (Wu et al. 2013; Yoshimi et al. 2014).

## CRISPR application to develop resistance against malaria by editing DNA of mosquito

Scientists have used the CRISPR system to create mosquitoes which can transfer resistance to malaria within the same species. For this, CRISPR was used to modify the DNA by injecting it into female Anopheles mosquitoes, which is one of the major parasite carriers in tropical areas and most notoriously in the regions of sub-Saharan Africa and Asia. The transmitted DNA is programmed with edited antibodies that could attack the malaria parasites (Gantz et al. 2015). This technique is still very new and requires further analyses and experimental designs before application. It is important to validate all the risk factors before its extensive use in nature. Once safely tested, it would be a great tool in the fight against fatal malaria. This application has shown 99.5% promise of in vitro mattings between modified and unmodified mosquitoes (Ghorbal et al. 2014; Hammond et al. 2016).

## Application of CRISPR in the treatment of HIV infection

Liang et al. (2016) of McGill University AIDS Center in Montreal, Canada applied CRISPR to delete the viral DNA that had been integrated into the human host cell by repair mechanisms. These studies have shown promises that genetically repaired tissue could be generated to prevent viral DNA function. Khalili et al. (2017) of Temple University in Philadelphia have used similar approaches by targeting multiple sites to reduce any chance for virus escape or the emergence of virus resistance to the primary treatment. Results of gene-editing CRISPR system to eliminate the HIV genome from the eukaryotic cells lines and success in the results have provided the potential of and promising strategies in treating HIV infection (Khalili et al. 2017; Ebina et al. 2013; Liao et al. 2015; Hu et al. 2014). Often when viral genome is edited, it could lyse the viruses, while in the other cases this manipulations could have the reverse effect by exacerbation of the viral infection in host cells (Lee and Lee 2019). On the contrary, if the viral repaired DNA takes an altered form, the CRISPR cannot identify and attack the host any more. This system could attack on various segments of viral genome and cause further alteration resulting development of higher resistance to available treatments (Wang et al. 2016). Alternatively, simultaneous the CRISPR-Cas9 system has been used for editing of the HIV co-receptors CCR5 and CXCR4 and considered it an hypothetically safe and promising approach to achieve treatment by protecting CD4(+) cells from HIV-1 infection (Liu et al. 2017; Allen et al. 2018; Kaminski et al. 2016). Particularly, few of current introduced systems may provide the opportunities and alternative potentials in current viral gene editing. A profound example of CRISPR application in human embryo was done by He et al. (Unpublished). He used 16 embryos with CRISPR and implanted 11 edited embryos into the wombs of women to attempt to create a viable twin pregnancy (Unpublished). He aimed to edit the gene to prevent the HIV in unborn twins (Lee 2019; Li et al. 2019).

## Medical applications of CRISPR for personalized use

**Fig. 3:**
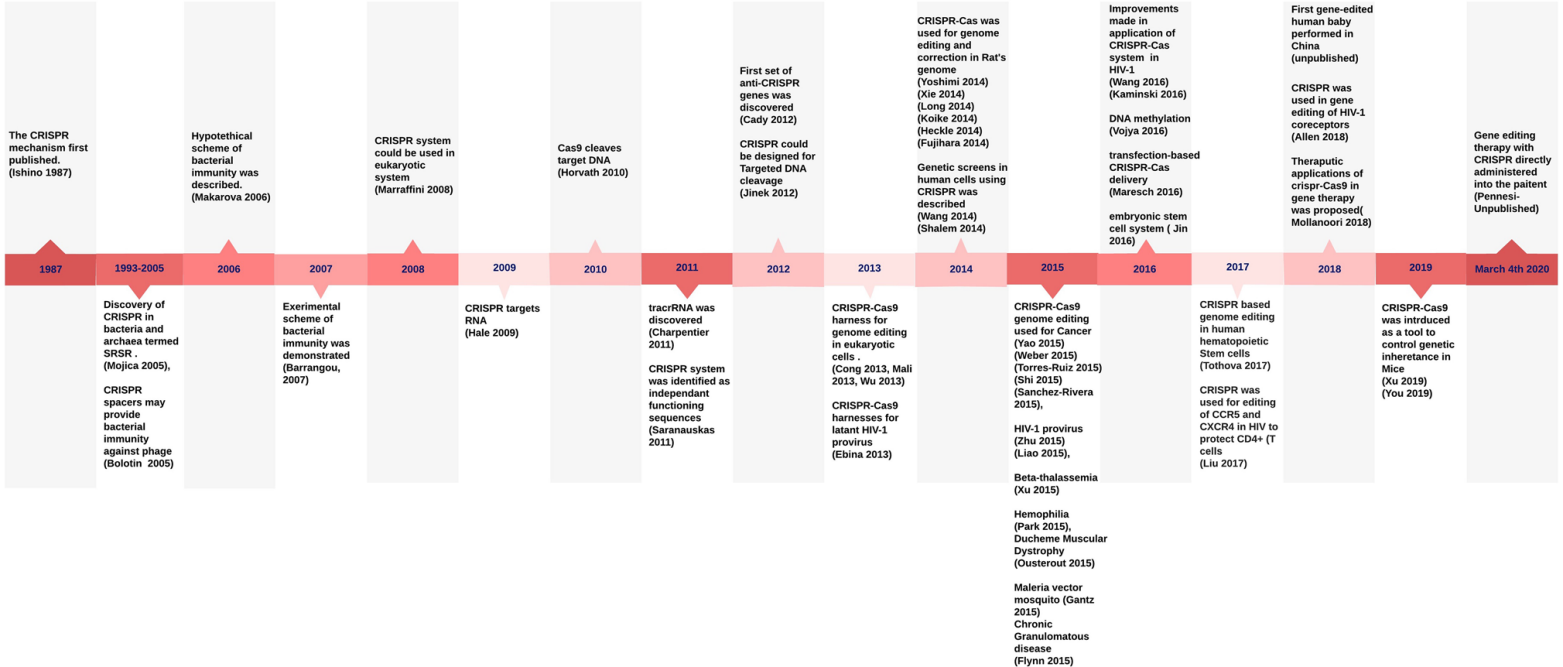
Timeline of key events in discovery and application of CRISPR system

Another potential application of the CRISPR technology could be in personal medical use, e.g.; embryonic stem cells would be engineered with the CRISPR system, and then, modified ES cells would be re-injected into the patient. For this, prospective patients are characterized based on their genotype and presence or absence of targeted faulty genes. These studies are very preliminary and are largely depends on the first testing or whether this is safe for the patient. For example, RuvC domain recognizes and cleavage the double-stranded DNA that is not complementary to gRNA, then active site catalyzes the single-stranded DNA following general two-ion of HJs (Sundaresan et al. 2017). By designing a mutation in the the RuvC Nuclease domains of the CRISPR system, scientists are planning to construct a engineered enzyme with lack of endonuclease activity and this, will be applied simply to facilitate identifying other targeted enzymes (Chen and Doudna 2017). Mutated *Cas9* causes the target protein to lose its endonuclease property but reserve its capability to recognize its DNA in a desired targeted region to bind to the excising sgRNA sequences. While technology will be used to enhance the binding of more enzymes to *Cas9*, to bind this complex to the desired target site-*Cas9,* an increase in enzymatic activity will be occurred to eliminate the cleaving of the sequence (Safari et al. 2019). Scientists are also looking for more clues to whether they might be helping patients in need since new Cas9-based tools are still poorly explored. In a study conducted on *SpCas9*, the results of a modified *Cas9* show that improvement of protein functions over domain fusion or splitting, rational scheme, and directed evolution is promising. Such protein engineering approaches proved to be more dynamic in the manipulation of DNA of organisms of the most interest, with safer precision, more accurate interaction with stimuli, and reduced or lack of toxicity, and more efficient application. In addition to engineered *Cas9*, sgRNA engineering has been developed to improve CRISPR application, and both techniques have enhanced the tailored *Cas9* tool applications. Cas9 could be used for simple or multiplex approaches for different genome editing and gene therapy. Epigenetic studies have shown reversible property of epigenetic correction, comprising DNA methylation, which has been now being used in cancer therapy for altering the epigenetic field (Shalem et al. 2014). Epigenetic editing at specific loci is another great approach for gene expression, and the CRISPR/Cas9-based approach has been developed for a precise DNA methylation, comprised of *dCas9* nuclease and catalytic domain of the DNMT3A. This tool targets gRNA of DNA and consists of CpG methylation segment. Once gRNA targets the dCas9-DNMT3A, several adjacent sites will be created, which enables methylation to increase in larger amounts and results in promoting greater increase of the direct DNA methylation in the sequence region of the target loci *IL6ST* and *BACH2,* eventually decreasing their expression (Vojta et al. 2016; Lei et al. 2018; Josipovic et al. 2019) (Fig. 3).

## Ethical factors for therapeutic application of CRISPR

As CRISPR and gene surgery could be a valuable approach in germline editing, these technologies grow more in scope. Since changes to germline could be passed on to the next generations, using the CRISPR tool has raised several social and ethical interests over its application, and these powerful tools deserve greater considerations (Walani and Biermann 2017; Brokowski 2018; Colnaghi et al. 2011). It is a fact that seven million children are born with lethal genetic disorders every year (Liu et al. 2015). The promising use of CRISPR as a tool for gene editing to treat defective unborn children, involving the success and accessibility of diagnosis and design, the treatment has generated debates among researchers to propose ethical guidelines for approval of this approach in preclinical settings (Curtis 2011). A survey conducted in 2018 of over 2500 Americans (Montoliu et al. 2018) showed general knowledge and acceptance of the idea for gene surgery in human embryos. While the vast majority of the USA population are not in favor of vaccination, blood transfusion, in vitro fertilization and organ transplant to save lives, it is difficult to press the issue to support the potential application of CRISPR in mankind to treat serious diseases. The public’s general knowledge is highly influenced by media, the idea of “barcoded humans, supper soldiers, or designer babies” does not represent the extensive clinical research in this field and requires clear vision for public (Silverman 2018; Jasanoff and Hurlbut 2018). Scientists equipped with this powerful technique should not use it as an enhancement tool for different human characteristics. Scientific communities, advocates, and ethicists should review ethics diligently, (Jiankui et al. 2018; Brenner 2002) based on personal ethics, religious beliefs, cultural values and public-health challenges. This requires debating and developing exclusive national and international safe guidelines for its unique applications in clinical trials (Silverman 2018; Regalado 2015; Nelson et al. 2016; Brokowski 2018).

## Conclusion

Previously, genome manipulation of humans was a theoretical concept; now, however, with the CRISPR technique we are taking noticeable steps to make that dream a reality. The evolution of every cutting-edge tools and technologies is crucial for scientific advancement. Nobel laureate Sydney Brenner is quoted as saying, “Progress in science depends on new techniques, new discoveries and new ideas, probably in that order” (Mollanoori and Teimourian 2018). It’s incredible to see how rapidly CRISPR research development has progressed, and this fascinating progress in the development of diverse range of CRISPR-based approches in just 20 years has shown promise in therapeutic applications, which is exciting for biomedical researchers (Baltimore et al. 2015). The CRISPR technology makes it promising to change genomes of nearly every organism under any conditions and continues to improve in a very precise, programmable platform in science for different biological and translational applications. It has become more convenient, cost effective, and well adapted in labs and biotechnology companies. CRISPR has increased our knowledge of DNA regulation and organization in every living cells across diverse species and continues to transform molecular biology, medicine, and biotechnology (Doench 2018; Funk and Hefferon 2018). For scientists in the CRISPR field of research, development of this system has led to the transformation of genome editing and has provide the great opportunities for therapeutic uses. Research labs are finding new applications in biomedical the engineering field and are exploring more purposes compared with other technologies. Progress in the development and understanding of CRISPR has revolutionized the field of medicine by specifying the demand for this technology in the prevention or the treatment of majority of genetic diseases (Hsu et al. 2014). CRISPR medical applications are developing and focus on treatment of cancer, HIV and other genetic disorders are on top list of clinical researchers, e.g. eradicating genetic defects in human embryos by using the CRISPR technique (Regalado 2015). The major obstacle for therapeutic use of CRISPRs in humans would be the correct delivery of editing reagents for effective gene correction in vivo or in stem cells that could be exploited to re-introduced into patients (Park 2016). However, in 2015, David Baltimore along with other researchers, ethicists and advocates commented that germline-engineering raises the risk of unanticipated consequences for future generation since there are limits to our current understanding of gene-environment interactions in human disease, genetics, and the pathways to other respective disease (Baltimore et al. 2015). He et al. (Unpublished) announced that his lab used the CRISPR technique to create genetically modified babies. Despite the ambitions about the technology which allows researchers to take such risks to make precise modifications to human DNA, (Stein 2018; Li et al. 2019; Barton and Rochman 2017) and like any great scientific inventions and discoveries, CRISPR approach has triggered ethical concerns among scientific communities that are not easy justifications. It is a fact that CRISPR is an exciting yet challenging area in modern biology with the aim of making notable change in the genome of different species, and germline engineering could be done exclusively on faulty genes that lead to severe diseases while there are not may practical therapeutic options available. In parallel to the current developments, extensive evidence on risk factors, and health benefits, improvements during clinical trials are required. Despite all its promises, this technique still requires crucial steps toward maximizing the system until it is considered safe enough to be used on humans.
